# A conceptual analysis of system responses to the issue of problematic sexual behaviors in children and recommendations for future work in Children’s Advocacy Center multidisciplinary teams

**DOI:** 10.3389/fpsyt.2023.1266463

**Published:** 2023-11-09

**Authors:** Mary Harris, Diane Lanni, Sasha Svendsen

**Affiliations:** ^1^Department of Pediatrics, UMass Memorial Children’s Medical Center, Worcester, MA, United States; ^2^Department of Pediatrics, UMass Chan Medical School, Worcester, MA, United States

**Keywords:** problematic sexual behavior, Children’s Advocacy Center, multidisciplinary teams, interdisciplinary teams, liberation health model, community response

## Abstract

Problematic sexual behavior (PSB) in children is a common, yet frequently misunderstood and mishandled issue facing communities. Because of the intersection of children both causing harm and being harmed, societies across the globe struggle with whether to punish or support during these times. For Children’s Advocacy Centers (CACs), whose mandate it is to support victimized children, this tension is exacerbated. CACs have historically relied on identifying a “perpetrator” and “victim” when providing their services, however PSB displaying youth do not fit this classic dichotomy. Compared with other children, PSB displaying youth are more likely to experience greater incidents and types of violent childhood trauma, have increased parent instability, decreased familial support, and struggle with co-occurring mental health diagnoses. Due to the stigma and fear surrounding sexual behaviors in children and systemic barriers including varied definitions of PSB, uncertainty regarding how to respond within the context of child-serving roles, and the criminalization of children’s behaviors, access to supportive services is complicated and challenging. Treatment completion rates for this population are as low as 13%, despite most methods being short-term, non-invasive, and community based. This conceptual analysis paper identifies five key themes in the literature that influence these barriers and proposes an interdisciplinary approach for CAC multidisciplinary teams (MDTs) to better support this vulnerable population.

## Introduction

Sexuality and sexual behaviors are common and expected aspects of child development. However, the diverse ways and contexts in which these behaviors are displayed, coupled with the stigma and bias surrounding these variables, make defining what is “typical” versus “problematic” challenging (1–3). Problematic sexual behavior (PSB) is generally defined as a behavior displayed by children or youth that involves sexual body parts or acts, is outside their expected developmental stage, and causes harm to self or others (2, 4). These behaviors tend to be minimally responsive to adult redirection, involve negative emotionality such as feelings of fear, shame, or anger, occur between youth of disparate ages, sizes, or abilities, and can use force or coercion to involve other children in the behavior (4, 5). It is helpful, therefore, to consider sexual behavior in children along a continuum.

Some studies have found that as many as 80% of youth will engage in some form of sexualized play or interaction with a similarly aged peer by the time they reach adolescence (2). It is also estimated that approximately 25% of cases referred to Children’s Advocacy Centers (CACs), and over one third of all cases referred to law enforcement, for concerns of sexual harm or misconduct involve youth acting out against other youth (3, 6–8). Given the tension between the commonality of children engaging in sexual behaviors during childhood and the serious implications of being labeled as causing sexual harm, it is important that professionals and communities critically evaluate these behaviors and assess how best to respond.

Most PSB responses are siloed into either the legal or mental health systems, with little regard for the family’s perspective in this process (9, 10). However, problems arise when these cases are not approached from a more holistic perspective. As few as 13% of youth referred for PSB treatment ever complete their intervention, despite recidivism rates for short-term PSB treatment being as low as 2% (3, 5, 11, 12). Drawing from the liberation health framework, this discrepancy is likely reflective of issues related to historical oppression and stigma driving siloed PSB responses (13, 14). Rather than communities critically analyzing their beliefs around sexuality, reflecting on how this impacts their perceptions around addressing PSB in children, and emphasizing their strengths as a diverse and interconnected system, they continue to draw from much of the same flawed and limited perspectives.

This paper seeks to address this issue. The authors begin with a review of the historical ways in which communities have responded to PSB to provide a better contextual framework. They then discuss five key concepts identified in the literature which have facilitated harmful or ineffective practice. Lastly, the authors offer considerations for future PSB response in communities, highlighting the unique role of CACs as a critical, interdisciplinary team that is well-positioned to address and respond to this complex issue.

Note: Throughout this paper, readers will observe that the authors use the term “parent” rather than “caregiver.” Drawing from the lived experience of one of the authors, while “caregiver” is often viewed as a more inclusive term for the various ways an adult can care for and raise a child, it can also be experienced as a way of othering and distancing non-biological parent–child relationships. Thus, the authors have opted to use the term “parent” to describe any person in a parenting role with a child. This includes, but is not limited to, biological parents, grandparents, aunts/uncles, and foster parents.

## A review of historical responses to PSB

Examining the historical context of PSB response is vital to ethical and clinically sound practice. Although helping professionals intend to do no harm, the reality is that providing help for complex and potentially stigmatizing issues is difficult to do. Intersections of belief systems, identity, systemic oppression, and vulnerability all intertwine with help and harm, and helping professionals must critically reflect on how they contribute to this process (14–16). It is important to recognize that, historically, PSB concerns were influenced by white supremacy, homophobia, heterosexism, and firm gender binaries. Professionals and teams must recognize the impacts of these origins and how communities conceptualize this issue so they can avoid inadvertently continuing it (13, 17–21).

### 1940s–1950s

Studies involving sexual behaviors in children first emerged in professional literature in the 1940s. “Appropriate” sexuality was defined almost exclusively by dominant western European, Christian values (13, 21–23). People who displayed overt sexual behaviors, were attracted to individuals of the same gender, or dressed or acted outside of ascribed gender norms were labeled as deviants who were too “ill” or “dangerous” to live in society (18, 22, 24). This belief was furthered by the first publication of the *Diagnostic and Statistical Manual of Mental Disorders* (DSM) in 1952, which labeled homosexuality as a form of “Sexual deviation” and placed it under the same diagnostic category of as “transvestism, pedophilia, fetishism and sexual sadism (including rape, sexual assault, mutilation)” [(25), p. 39; (26)]. Sexuality and diversity were both seen as threatening to the safety and morality of a community, and those who did not align with what was set forth by society were subjected to harmful treatments, institutionalization, and even criminal charges (1, 22, 27).

### 1960s–1970s

During the 1960s and 1970s there was a pivotal societal change that allowed the diverse spectrum of sexuality to be considered. Writings like the ‘Kinsey Report’ argued that all people—including women and children—experience a range of sexual behaviors and experiences, and professionals were challenged to better define what constituted a “typical” versus “concerning” behavior (27–30). With this growing understanding that children were sexual beings, fear and questions arose about the connection between PSB and adults who sexually harm (24, 30, 31). However, rather than addressing these questions, communities responded by either institutionalizing youth displaying PSB for indefinite periods of time or ignoring the issue altogether in hopes that such behaviors were a “phase” the child would grow out of (24, 32). There was little thought or regard for the long-term implications of this response, and present day research shows that this type of practice ultimately placed youth at greater risk of both PSB and future harm (6, 33).

### 1980s–1990s

Due to the long history of the punitive response to PSB through the legal system, families were increasingly fearful and wary of seeking help from professionals (34, 35). Youth with PSB were labeled as “super-predators,” which resulted in stigmatization of this population (1, 7, 36). This made it difficult for researchers and practitioners to obtain accurate data to guide their decision making (34, 37). Rather than having reliable, longitudinal data from the children and families struggling with PSB to inform care, professionals were forced to rely on arrest and court records that were severely limited and inaccurate (35).

Professionals came together for the first time in 1987 to address these challenges, and they created a national task force to address PSB in children (38). The result was a unanimous call for better research and a more structured response to support treatment efforts (35, 37). With improved assessment and response tools, clinicians and advocates for this population could begin to identify risk factors for PSB and collect more accurate data to support their claim that treatment with children could be successful (34, 35, 39).

### 2000s

Although the history of PSB was marked by fear that these youth were doomed to become adults who caused harm, the research of the 2000s presented a very different picture. It was demonstrated that youth who struggled with PSB were more likely to be victims of violence themselves and to have co-occurring difficulties in the areas of emotion regulation, social skills, and other mental health diagnoses (6, 40, 41). Furthermore, the types of sexual behaviors displayed were vastly diverse, which meant that addressing the child’s needs and treatment responses had to be individualized to the child’s unique context (2, 36, 42). Treatment that specifically engaged the family unit was shown to have positive results, with recidivism rates ranging between 2 and 10% (40, 43, 44).

### Present day PSB response and the role of the CAC

Professionals and communities continue to work to address PSB in a variety of ways. Because research emphasizes the importance of responding to PSB in a clear, unified, family-centered manner, CACs are increasingly identified as a logical entity to facilitate this work (3, 10, 45). There are over 1,100 CACs around the world designed to keep children safe and centered within an MDT response (10, 46, 47). Teams of professionals, including law enforcement, District Attorney staff, child welfare workers, medical providers, and mental health professionals, all come together to ensure that the needs of vulnerable children are met in a way that does not cause further trauma or confusion. However, one of the challenges in addressing PSB concerns in this setting is that CACs were developed from a “victim/perpetrator” framework. Youth with PSB do not fit into this classic duality, and therefore, a different approach is needed.

Recent international CAC PSB research shows that teams often feel they are being “disloyal” to the child who is the recipient (e.g., the “victim”) of the PSB behavior if they provide support to the displayer of the behavior (e.g., the “perpetrator”) (3, 45). However, attempting to classify the children in this way can do more harm than good. PSB in children is a very different issue from child sexual abuse perpetrated by an adult, and children who struggle with PSB respond well to education, positive relationships, behavioral modifications, and treatment (48–50). Given that a great deal of PSB occurs within family units, typical responses of simply separating the children is not only difficult to do, but it often causes additional harm for both youth because the child who is the recipient of the PSB often feels a sense of guilt and loss over their sibling’s removal (3, 45, 50).

Recent literature supports the ways in which CACs can address these gaps. In 2020, Sites and Widdifield published a white paper report titled, ‘Children with Problematic Sexual Behavior: Recommendations for the Multidisciplinary Team and Children’s Advocacy Center Response’ (10). In this report, the authors highlight the strengths of the CAC model and how multidisciplinary teams can support all children involved in issues of PSB. They suggest that, with small changes to pre-existing protocols, increased mental health provider involvement, and inclusion of families in the conversation, CACs can continue their work of supporting all vulnerable children (3, 10).

## Key concepts

Throughout the authors’ cumulative experience working with families, they repeatedly identified several challenges when addressing PSB. To better understand this phenomenon, they conducted an extensive review of historical and present day literature, ranging from 1943 to 2023. This served to validate the authors’ observations, and the authors subsequently categorized these reoccurring themes into five main concepts: Difficulty defining PSB, use of short-sighted safety responses, disregard for the intersection of PSB with other needs, lack of parent involvement and engagement, and siloed responses. It is important to note that these identified gaps and challenges, while difficult, also provide opportunities for CAC MDTs to improve service delivery to this special population.

### Difficulty defining PSB

Attempting to define problematic or harmful sexual behavior is a difficult task. Although it is widely accepted that PSB is a sexual behavior displayed by a child that is outside their expected developmental trajectory and causes harm to self or others, there are a multitude of nuances that make defining this problem challenging (17, 51). Factors like whether the displaying child has reached puberty, the age of the other child(ren) impacted by the behavior, the type of behavior displayed, any use of additional forms of violence, and parent attunement and response (2). Furthermore, adults tasked with protecting children often struggle with their own emotions and sense of safety related to childhood sexuality and sexual behaviors, which contributes to the challenge of establishing a clear and unbiased definition of PSB (1, 9, 42).

Despite these complexities, one of the most common ways PSB is categorized is by distinguishing between whether the youth displaying the behavior is categorized as a “child” (a youth under the age of 12 years) or an “adolescent” (12 years or older) (3, 17, 51). The impact of puberty, sexual gratification, and the desire for sexual relationships are important influencing factors in both the displaying of PSB and its treatment in adolescence (2, 33, 50). Societal factors, including the role of the legal system and risk of prosecution are also key influencers behind the push to use age as a component of assessment (3, 52).

Studies have found that a state or country’s legal age of consent and/or prosecution influence, if not determine, whether support is given to a child (3, 7, 52). Although age allows for clearer definitions in matters of the law, communities are cautioned against placing too much weight on this one factor when assessing level of risk or severity related to PSB (33, 51). Instead, communities are encouraged to think more critically about PSB and to use individualized, developmentally based assessment tools that can provide context to the behavior and the child (9, 42, 43). This not only provides teams with a deeper understanding of the factors influencing the development of PSB, but also provides valuable insight in how to address and respond to it.

### Use of short-sighted safety responses

Historically, most PSB interventions focused on physically separating the displaying child from their families and communities. Because these youth were viewed as a potential threat to society and to other children, heavy emphasis was placed on ensuring safety in the most clear-cut way possible (13, 19, 36, 53). This included removing the child from their home environment and placing them in juvenile justice or residential treatment facilities, and/or placing them on sex offender registries. However, there are long-lasting and potentially devastating consequences to this approach.

Research demonstrates that use of sex offender registries and “dishwasher” treatment—or treatment where a youth is removed from their environment to be “fixed” and returned to their original environment upon “completion”—is not only ineffective but harmful (1, 6, 19, 53, 54). Placing children in residential facilities that have higher concentrations of youth struggling with PSB and other significant needs, and decreased access to parental supports and connections, puts them at higher risk of both future victimization and continued displays of PSB (6, 33, 55). While some children require intensive, inpatient treatment to address highly intrusive and violent displays of PSB, most youth do not fall into this category. Most youth respond well to short-term, community-based therapy that includes and supports parents in the safety planning and behavior modification process (10, 17, 50). Additionally, because of the high level of success with therapy, placing youth on sex offender registries which follow them well into adulthood has been found to do little more than exacerbate challenges for the youth and their family (19, 36, 53).

Conversely, working with the family to assess and respond to PSB concerns allows teams to better understand the concerns and behaviors within the context of the child’s environment and to address them in a more timely and effective manner (44, 48, 50). This approach also provides insight into the family’s protective factors, which can be used to further support the child and ensure that safety needs are met. Because PSB is frequently rooted in trauma and relationship difficulties, working to support the child within the context of relationships has immense value (45, 48). Therefore, when teams decide to isolate children from, or even within, their environment, they must critically examine the implications of such a choice. While this may provide an immediate, short-term solution to PSB in the community, research suggests it does little to support long-term safety and healing for families when done outside of a comprehensive and developmentally sensitive manner (7, 36, 53).

### Disregard for the intersection of PSB with other needs

Children who struggle with PSB often have additional intersecting needs that make supporting them both important and challenging. One of the earliest intersections identified in the literature is the connection between PSB and prior victimization (2, 17). While it is important to note that upwards of 95% of children who have experienced sexual abuse do not go on to display PSB, youth who display PSB are significantly more likely to be victimized in this way (6, 9). Furthermore, research suggests that a child’s risk for PSB increases with the number and types of victimizations a child experiences—particularly when violence is involved, as with physical abuse and domestic violence (33, 52).

Youth placed in foster care or congregate care settings, or who are involved with the juvenile justice system, are also at greater risk for both displaying PSB and being impacted by the PSB of other youth (6, 9). One possible reason for this is that youth in care are less likely to benefit from protective factors like parent connection and guidance. Attuned parents can both alleviate trauma symptoms and provide supervision when concerning behaviors are identified, both of which are key in addressing and preventing PSB (2, 33). Thus, the compounding factor of early childhood trauma and reduced parental protection and support increases the likelihood of PSB in children (6, 33).

Youth with PSB are also more likely to struggle with co-occurring behavioral and mental health difficulties. Issues related to social and emotional awareness, impulse control, and self-regulation commonly intersect with PSB (2, 9, 17). Therefore, treatment responses must be comprehensive in their approach (10, 43). They should include a combination of psycho-sexual education, social skill building, self-regulation techniques, and trauma processing (33, 50, 51). Likewise, responses that are interdisciplinary in nature—spanning the boundaries of family members, educators, mental health providers, and legal and medical systems—have also been found to be beneficial in addressing the complexities of this population (3, 7, 12).

### Lack of parent involvement and engagement

The impact of parents on PSB is well documented in the literature. Parent involvement and responsiveness has been found to be one of the key protective factors in both the development of PSB, as well as in promoting successful treatment outcomes (12, 48, 51). Therapeutic responses that include parent skill building around behavior management, boundary setting, and communication were found to be among the highest predictors of successful treatment outcomes (2, 3, 44, 50). This suggests that empowering parents in their ability to both address the behaviors and improve their relationship with their child are mutually beneficial to addressing the problem of PSB.

However, psychosocial influences of fear, stigma, guilt, and generational trauma have powerful influence over a parent’s receptiveness to discussions of PSB and safety planning (12, 48). Because PSB involves harm to a child, a child welfare report and District Attorney referral are often made following any disclosure or discovery of PSB (3, 10, 45). The report filed to the child welfare office is typically documented as a concern of ‘parental neglect’, and the referral to the District Attorney is typically for allegations of ‘child sexual abuse.’ Although there are important reasons behind these protocols, including the need to ensure the safety of children and to connect families with emergency assessments and support, this experience frequently leaves families feeling far from supported or empowered.

Parents often report feeling judged, confused, powerless, and isolated following a system response to PSB (12, 45, 48). Rather than families finding the support and clarity they need from their interactions with child-serving professionals, they frequently experience these agencies as a threat to their family and to their child’s future safety. This results in increased defensiveness and resistance to engagement and provides valuable insight into recent research findings which demonstrate that treatment completion rates for this population are as low as 13%, despite even lower recidivism rates (11, 12).

One way the literature suggests addressing these challenges is by consciously partnering with parents and including them in the PSB response process (2, 48). This allows parents to better understand the issue of PSB and provides them with tools to address it (10, 50). This approach also increases trust between the families and the professionals positioned to help them. Community multidisciplinary teams should draw from the growing body of literature which has demonstrated PSB treatment to be highly successful, and therefore can provide families with a sense of hope for the future. Because of the level of stigma continuing to surround issues of PSB and child sexual behaviors, teams must be willing to explore a family’s fears and challenges in order to build an open, trusting, and collaborative working relationship.

### Siloed responses

Because PSB is a unique and complex issue, a diverse group of perspectives is required to address it. As early as the 1960’s, PSB practitioners have leaned on the resources and skills of their colleagues in other fields to help support youth who display PSB with great success (24). For example, one single outpatient therapist could not feasibly provide weekly treatment, assess safety in the home, and ensure families followed through on all recommendations put forth by the courts. However, in partnering with local probation officers, the therapist could remain in the role of mental health practitioner, knowing that various aspects of the family’s needs were being met by other professionals. Furthermore, in working collaboratively, the therapist received vital information from the probation officer about how the family was doing and whether progress was being made. This benefit was reciprocal in nature, as the probation officer also benefited from the therapist’s clinical opinion regarding the child’s progress in treatment. Thus, children and families were better served through this collaborative and integrated approach.

CACs are a prime example of this collaborative approach. CAC MDTs meet regularly to ensure that all team members working with a family have the same information regarding concerns identified, steps taken to ensure safety, and next steps needed to support the child (10, 45). While this is undoubtedly beneficial in ensuring clear communication between partnering teams and systems, there are limitations to this way of practice. MDTs maintain distinct boundaries around their roles and communication with one another. Each discipline speaks to their own work, and typically has unique goals and agendas related to their professional role in a child’s case (56). For example, law enforcement and District Attorney team members focus on upholding their role as investigators and prosecutors of crimes against children, whereas mental health providers focus on their role in providing ongoing support and treatment. While each role has valuable contributions, in isolation, they do not accurately reflect the whole picture.

It is the intertwining of interdisciplinary perspectives that contributes to optimal outcomes. Rather than having separate goals amongst the MDT, interdisciplinary teams strive for a shared common goal and purpose (57, 58). While this has the potential to result in conflict and disagreements amongst team members, the interdisciplinary framework acknowledges this shared approach as a means of ensuring that issues are being addressed in a holistic manner (56, 59, 60). Rather than teams remaining siloed, with their own values and biases, they are pushed to deconstruct their ideas and see what help or hinderance they provide to the process and to the family.

To assist in this process, interdisciplinary practice emphasizes inclusion of the lived experience perspective. Lived experience offers teams invaluable insight into the issues that their clients face and bring to light any barriers or challenges that arise (9, 54, 61). It also helps teams to address the ongoing challenge regarding the stigma of PSB and the difficulty of families to trust and engage in the process (12, 48). Through access to people who have previously been through the process of PSB identification, response, and treatment, families can be reassured that healing is both real and possible.

## Considerations for an initial support and stabilization response for CAC MDTs

In light of these five key concepts, the authors offer three perspectives for CAC MDT members to consider when responding to initial PSB concerns. Rooted in the liberation health framework, the authors seek to demonstrate how their own interdisciplinary collaboration has helped to support families and one another during a time of PSB response, which has been shown to be instrumental in determining whether families successfully engage in PSB community supports (10).

Drawing from the medical provider perspective, Child Abuse Pediatrician Dr. Sasha Svendsen suggests a role for the medical provider within the CAC initial response to assess the behavior in the context of typical childhood sexual development, which not only helps to decrease the stigma associated with this topic, but also allows for a more neutral space to explore and reinforce body safety, body autonomy, and healthy boundaries. Drawing from the social work perspective, clinical social worker Dr. Mary Harris discusses how CAC MDTs can develop on-going critical dialogue and reflection to improve awareness of the biases and silos impacting family engagement and successful PSB outcomes. And finally, drawing from the lived experience perspective, parent and peer support professional Diane Lanni shares her experience as a caregiver of multiple youth who have struggled with PSB and as someone who has engaged with child-serving systems to address it. Ms. Lanni discusses the power of humanizing the issue of PSB and including families in the response process.

### Theoretical framework

The liberation health framework is a radical social justice theory that sees value in bringing groups of people together to tackle difficult problems (62–64). Rather than certified “professionals” being seen as the experts on a person or community’s situation, the liberation health model posits that all people—particularly those with lived experience—have important knowledge to contribute to fully understand and explore issues (14, 15, 65). In working collaboratively, better solutions come about, which leads to a more just, equitable, and healthy society (62, 64, 66). In drawing from a group of diverse perspectives, the entirety of the problem can be better defined and addressed.

Reflective of social work’s person-in-environment perspective, the liberation health framework acknowledges that the issues people face, as well as their strengths, occur within an important context (14, 64, 67). Although people are unique individuals, they exist within a larger historical and societal framework of intersecting identities and structural forces that impact their ability to succeed or struggle in life. Seeing issues in this way allows for recognition that inequities, oppression, and a variety of -isms have significant influences and far reaching impacts (15, 64, 68).

In the case of PSB, the liberation health model helps to frame things like the discrepancies between the high rates of incomplete treatment and low rates of recidivism following treatment as a symptom of larger structural and societal barriers. When powerful child welfare and protection systems exert their dominance by problematizing the child and using threats of removal and legal charges to force families to act, rather than acknowledging the influences of trauma, prejudice, and lack of community resources on PSB development, families cannot help but respond in fear and retreat (19, 64, 69). Rather than the family and system coming together to critically examine how PSB concerns came about, the groups become siloed into opposing camps. This ultimately negatively impacts both the discovery of the problem and its resolution because, according to the liberation health framework, there is a connection between the issues and the solutions (14, 63, 64).

The authors drew from the liberation health framework as a way of contextualizing the challenges of PSB and providing a way forward for communities. It was important to choose a theory which would not only provide insights into the challenges of PSB—a daily reality for the authors—but that the framework would provide tangible and unique solutions as well. Early influence of this theory on this paper can be found in the authors’ decision to perform a chronological literature review. Liberation health purports that complex social justice issues are often rooted in historical oppression, and gaining an awareness of that fact is a crucial first step to understanding how to address it (15, 63, 66).

Because liberation health posits that issues and solutions go hand in hand, the authors also utilized this framework as a way of deconstructing the five key concepts/challenges identified in the literature and the authors’ experiences to find solutions. If isolation and siloed power lead to issues of unjust PSB response and continued barriers to support and treatment, then the solution to such things, according to liberation health, is inclusion, collaboration, and empowerment (15, 63).

In the final section of this paper, the authors draw from this theoretical framework to provide “considerations” for future practice rather than “recommendations.” The liberation health framework posits that a key feature of liberatory practice is that people and communities must be empowered to think and make choices for themselves and their own unique context (14, 15, 66, 68). This is also supported in the PSB literature. PSB and the families and children who struggle with it are diverse and have unique needs and strengths. A “one size fits all” model often does more harm than good. This framework allows the authors to protect against such things, while still pushing communities and professionals to take actionable steps toward change.

### Considerations from the medical perspective

It is important to understand that sexual behavior occurs along a continuum, ranging from typical and expected to abusive and violent. When determining whether or not a sexual behavior is problematic, there are generally three factors to consider that can help to characterize the behavior along the spectrum. They are: (1) the frequency of the behavior, (2) developmental factors involved, and (3) the level of harm to all the children involved (2, 17). Furthermore, as previously noted, the relationship of the behavior within the context of the child’s overall development and their environment must also be considered.

By (first) exploring this issue from the developmental perspective of the medical lens, this complex and often emotionally charged topic can be initially assessed in a more neutral, objective, and non-threatening way (2). Children and their parents have unique relationships with their medical providers, particularly if a medical home has been established for continued routine care. This is an ongoing, trusted relationship, in which providing anticipatory guidance to the parent about the child’s growth and development is paramount. Consideration of the behavior within the context of typical, expected sexual development and health provides a de-stigmatized and family-centered approach, which allows for various aspects of the behavior to be discussed in order to fully understand the context of the behavior being displayed (2). This includes exploration of any sexual abuse or other trauma history, exposure to sexually explicit content, as well as various parenting or cultural practices. By approaching this topic from the medical perspective, medical professionals can help children and parents to understand that sexual development consists of more than just hair growth and other physical body changes. This conversation allows an important opportunity for the medical professional to introduce and reinforce the concepts of body autonomy, body safety, boundaries, and healthy relationships.

When a child’s problematic sexualized behaviors are approached through a similar inquiry and triage process as other medical and developmental concerns, it allows space for honest dialogue and initial assessment, whereby the medical professional obtains initial information, asks clarifying questions, and ultimately decides on action plan based on immediate identified needs. By no means does this process replace the need for a more comprehensive assessment of the situation and treatment planning, it merely serves as an initial step to address and assess the concern at hand. It then requires coordinated follow-up with the community interdisciplinary team, where a more comprehensive assessment will be provided (10). By highlighting the role of the medical provider in the early triage process once a concern has been identified, the children who truly need the specialized, yet limited resources can be more accurately identified, as well as those children who do not. The impact of this first initial step would greatly impact the already overwhelmed and under-resourced mental health services available for children who display problematic sexualized behaviors (10).

Interactions with medical professionals, particularly during yearly well visits, has the potential to provide children and parents with a model for how to have conversations about body safety and body autonomy, which can then be reinforced between the parent and the child at home. These conversations represent a critical step in the early stages of the process, while the child and parent await the larger, more comprehensive assessment. This modeling of communication also serves to empower parents with an actionable step and provides them with a sense of agency while awaiting connection to longer-term services.

Initial supportive and non-punitive responses from medical professionals may help to overcome some of the barriers associated with this issue and will hopefully promote a sense of support and encouragement for parents to engage in additional communications with other interdisciplinary team members. The way in which the child-serving professionals of the interdisciplinary team frame and approach this issue with children and their parents is crucial for parent engagement in the larger process, which research has demonstrated to be the single most pivotal factor for a child to stop engaging in problematic sexual behavior and to support them to make more positive and healthy choices (10, 40). In all conversations with parents, this topic must be humanized and the context of the behavior must be considered. Everyone must understand that the behavior does not define the child or their future. By helping children and parents to understand that the behavior is the problem, and not that the child is the problem, children and families can develop resilience factors and promote a strengths-based approach to safer, more healthy choices in the future.

### Considerations from the social work perspective

Critical reflection and dialogue are important steps to addressing complex issues as a helping professional. Despite many good intentions, research shows this is not enough (19, 36). Social workers and other helping professionals sit in places of power over vulnerable clients, especially in fields like child welfare, so careful work must be done to ensure this power is not used to cause further harm. Professionals need to be aware of the many intersecting influences that impact clients and the concerns they face. Issues like bias, stigma, oppression, and other injustices are often invisible at first look, but immensely impactful on the lives of these youth (1, 19).

Intersections of children both causing and experiencing harm also leave many adults, both professional and not, feeling frightened and unsure where to turn. It is important for teams to critically examine why sexual behaviors in children trigger such intense reactions. Despite the relatively common experience of sexual play amongst children, and the knowledge that children respond well to clear and consistent designations of body and relationship boundaries, adults continue to struggle with their own perceptions and beliefs around what constitutes “expected” and “safe” behavior. This is problematic when thinking about PSB response because it places children at risk of being overly or erroneously labeled as having a problem, based on whether the behavior falls outside the professional’s own experience or set of values (1, 13, 42).

One way teams can combat this is by leaning on one another for collaboration and discussion. CAC MDTs were designed to support children, but they can also provide immense support to the adults working within them. In having a space to discuss cases and safety plan, MDT members can dialogue about their concerns and experiences, and receive feedback. This give and take of perspectives provides teams with a robust knowledge and understanding of the issues facing their communities (56, 57, 60). It also protects against the centering of one dominant opinion over another and helps teams to see the various factors that have led to the concern. For issues of PSB, this means teams can see their clients in a more holistic way, and ensure their needs and experiences remain the focus of the MDT response rather than the beliefs of any one team member.

However, for this type of critical practice to take place, it is important that all people involved in the PSB response be represented in the conversation—including the families. Although this can be challenging for some professionals because systems of power often work behind the scenes and in silos as a way of protecting themselves and maintaining control, including families and other perspectives in the conversation is vital (57). Parents provide unique and invaluable insight into the child and the struggles they are facing. By including families in the conversation, teams obtain a much clearer picture of what is happening and how to proceed (44). Creating a space of open and honest dialogue reduces the risk that families—and teams—feel like they must defend themselves against one another—one of the most common barriers facing PSB response. In seeing that all team members add value to the conversation and have a common goal of achieving health, wellbeing, and safety for all children, teams can make great strides in the work of PSB response and change the trajectory for this special population.

### Considerations from the lived experience perspective

PSB often occurs within the child’s home but regardless of where the behavior occurred, parents often feel responsible and fear the judgment of society (3, 12, 70). “What will people think?” is a common worry. Parents may hear folks say, “they learned that somewhere” and worry that not only will their child be judged, but their entire family and parenting will be called into question. Fear and shame make it challenging for parents to reach out for the help they need for their child or children. When parents are the caregivers to both the displayer and recipient of PSB, there are additional emotions and a concern for how to support each child (3, 45). The emotional toll is heavy, and families need support in real time to prevent further emotional harm to all family members. Systems, understandably so, are focused on the immediate safety of children, but for parents, psychological safety is paramount. The basic questions of, “Is it safe to tell?” and “Who is a safe person to tell?,” are at the forefront of most parents’ minds.

Family engagement begins with creating an environment where everyone feels safe to be open with discussing what occurred and agreeing to a safety plan (10). Yet, are all members of a multidisciplinary team safe? For parents, they worry that accepting help is an admission of guilt. Seeking help is frightening, especially for marginalized individuals who may already feel distrust with systems. Even when families bravely try to seek help for their children, many do not know where to go. “Who do I call?” “How much do I tell?” “Do I begin with the police or the hospital or a therapist?” “Is someone going to show up on my doorstep and take my child away?”

As mentioned, the barriers to engagement with parents are many, yet we know that parent engagement is critical for successful outcomes for children (2, 12). PSB by its very nature is personal, and since the damage occurred in a relationship, healing must occur in the form of healthy relationships. Families must be empowered as the agents of change. All family members should be a part of the collaborative effort to understand what has happened and agree to an action plan. Considering the emotional vulnerability of parents, it is prudent to design the team engagement with attention to parent needs.

One way to facilitate the psychological safety of parents is the use of a peer support person. Family members are more likely to trust information from someone who has been in their shoes. Use of peer supports for parents does more than just support the parent. A peer support person can act as a critical link for information between team members. Peer supports act as “cultural brokers,” as they are comfortable navigating in both professional and familial settings (71, 72). They can often express the chief concerns of parents in a way that promotes understanding and reduces shame and stigma. Peers assume the role of an emotional container for the parent, allowing the parent to process their own thoughts and feelings, thereby giving space for needed safety planning and communication with other team members.

The time waiting for professional help can been painfully long for parents. Knowing there are actions steps to take while awaiting services can help reduce the stress and anxiety of that time period. Can my child stay at home? How can I prevent a recurrence of harm? Who do I reach out to when new household rules are broken? Should we talk about what happened or should we wait in silence for the professionals? How do I educate myself and others about how to handle PSB? Who needs to know? What do I do if there are more disclosures or behaviors? Parents may be afraid to ask many of these questions and will need an empathetic ear on the team to help them bravely ask for help. Resources should be readily available to give to caretakers when these questions arise. Parents need to know they are not alone while awaiting therapeutic services in a way that recognizes everyone involved, not just the impacted child.

## Conclusion

Despite the issue of PSB being present in the literature for over 80 years, communities continue to struggle with many of its basic tenets. Defining what constitutes a behavior as problematic or harmful, effectively addressing the behavior, understanding the various intersecting factors that influence its development, including parents in the conversation, and valuing the power of interdisciplinary work, all coincide to make this a complex but important topic to consider and deconstruct. The liberation health framework offers a way to understanding this issue and to address it in a more holistic, inclusive, and socially just manner.

Through use of the liberation health framework, the authors critically examined the historical context of PSB and identified five key concepts and challenges communities face when attempting to address this issue. These challenges were then deconstructed and used to identify opportunities for change. The authors then offered their three, unique perspectives on how CAC MDTs can address this issue in their own communities. Reflective of the liberation health perspective, the goal of this paper was not to provide a concrete response model to be replicated by all. Rather, the authors focused on empowering communities and interdisciplinary professionals to find their own, unique way of responding to PSB concerns. In working in this way, the authors sought to ensure the issues of the past would come to light, be addressed, and result in long-term healing and thriving for all families.

## Author contributions

MH: Writing – original draft, Writing – review & editing. DL: Writing – original draft, Writing – review & editing. SS: Writing – original draft, Writing – review & editing.
